# Acute Life-Threatening Hemorrhage in Neonates With Severe Hemophilia A: A Report of 3 Cases

**DOI:** 10.1177/2324709618800349

**Published:** 2018-09-18

**Authors:** Alvaro Moreira, Hrishikesh Das

**Affiliations:** 1 University of Texas Health Science Center at San Antonio, TX, USA

**Keywords:** hemophilia, neonates, clotting factor, intracranial hemorrhage, splenic rupture

## Abstract

Hemorrhagic shock is a rare, emergent condition that is often fatal in newborns. In this article, we report cases of 3 neonates presenting with acute, life-threatening hemorrhage who were subsequently diagnosed with severe hemophilia (<1% factor VIII). The first infant was tachycardic, pale, and had a precipitous drop in his hemoglobin secondary to a subgaleal hemorrhage. The second patient sustained a splenic rupture, a sequela that has been reported in only 4 other neonatal cases. The last infant presented with tonic-clonic seizures and respiratory distress. Head imaging demonstrated extracranial and intracranial hemorrhage, complications that can result in 20% mortality. All 3 patients were successfully treated with clotting factor concentrate and blood products. After normalization of factor VIII levels, the newborns did not develop any new hemorrhages and were discharged home within 3 weeks of birth. Pediatric providers should be aware that these signs and symptoms may be potentially lethal complications in neonates with severe factor VIII deficiency.

## Introduction

**Figure 1.**
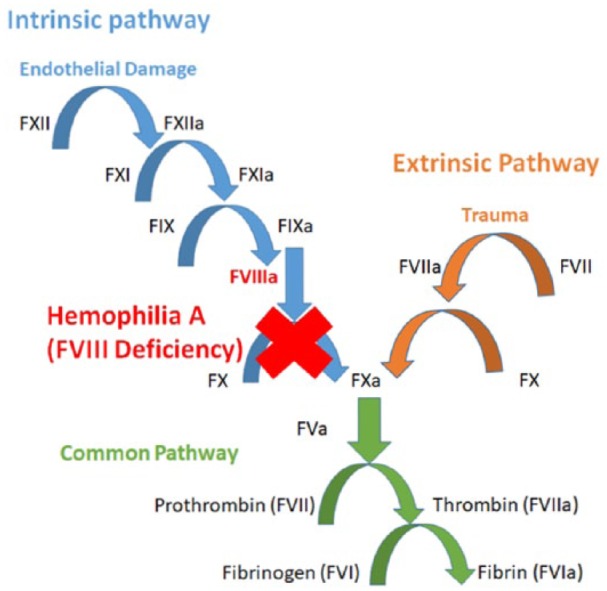
Complete coagulation cascade depicting both intrinsic and extrinsic pathways. Hemophilia A disrupts the intrinsic pathway and interrupts fibrin formation.

Hemophilia A (HA) is an X-linked recessive blood disorder characterized by decreased levels of coagulation factor VIII (FVIII). This deficiency affects 1 per 5000 males, disrupting the normal propagation of the intrinsic coagulation cascade. FVIII is a cofactor for clotting protein factor IX, and together, they form a complex that activates factor X.^1^ Any interruptions in this cascade have downstream effects, leading to an inability to convert fibrinogen to fibrin and resulting in clinical bleeding (Figure 1).^1,2^

Normal plasma levels of FVIII range from 50% to 150% (0.5-1.5 U/mL).

HA is categorized according to the amount of FVIII levels present: mild (5% to 50%), moderate (1% to 5%), and severe (<1%, or <0.01 U/mL). In some populations, up to 58.1% of those with HA had FVIII levels <1%.^3^

Patients with mild-to-moderate HA rarely incur spontaneous bleeding and often go undiagnosed until the age of 5 to 6 years.^4^ These patients will typically present with prolonged bleeding after minor trauma, different from children with severe HA who are diagnosed usually within the first 2 years of age. Children with severe FVIII deficiency have multiple episodes of spontaneous bleeding per month and can range from minor mouth bleeds to severe cranial hematomas from slight head trauma.^4^

In this case series, we describe cases of 3 neonates who sustain extracranial, splenic, and intracranial hemorrhage (ICH). The goal of this report is to illustrate the acute and potentially lethal clinical spectrum of HA in neonates. Up to 30% of patients with HA have a negative family history,^5^ and even those with severe HA have a median age of diagnosis of 5.8 months. Therefore, it is imperative that neonatal providers are cognizant of the different presentations of HA to prevent mortality and minimize morbidity.

## Case 1

A male infant, who was term appropriate for gestational age, was born via repeat caesarean section to a healthy 26-year-old woman. No family history of bleeding disorders was reported by the mother. Pregnancy was unremarkable, and Apgar scores were 9 and 9 at 1 and 5 minutes, respectively. On initial physical examination, a localized hematoma to the left parietotemporal region was identified. A routine complete blood count (CBC) displayed normal counts. It was decided to repeat the CBC in 12 hours and, in the interim, to observe for dissemination of the blood collection. Prior to the next blood test, the physician was called to the bedside because the infant now had a change in his clinical examination: pale and tachycardic. His examination now revealed a large palpable fluid wave that extended behind the neck and left ear. Given the infant’s change in clinical status and concern for a subgaleal hematoma, the infant was transferred to the neonatal intensive care unit (NICU). The repeat CBC showed a hemoglobin level of 7.6 g/dL, with a hematocrit of 22% and platelets of 169 × 10^9^/L. In the NICU, the patient received a transfusion of 20 cc/kg of O Rh-negative packed red cells. His coagulation profile disclosed a normal prothrombin time but an activated partial thromboplastin time (PTT) of 101 seconds (normal = 25-30 seconds).

Subsequently, a FVIII level was ordered, which was very low at <0.01 U/mL. He was diagnosed with severe HA and was initially treated with 50 U/kg of recombinant FVIII. His repeat doses were adjusted to bring his FVIII level to 100%. A computed tomography (CT) scan of the head revealed a large subgaleal hematoma, beginning in the left parietal area, extending to the occiput and down the nape of the neck. The infant was discharged at 1 week of age following a repeat head imaging confirming complete resolution of the extracranial hemorrhage (ECH) without any evidence of intracranial bleeding.

## Case 2

**Figure 2.**
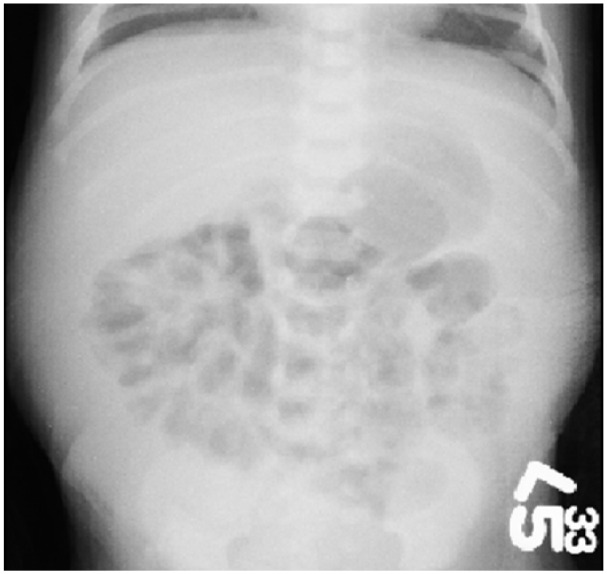
Abdominal radiograph, anterior-posterior view of infant in Case 2. The intestinal gas pattern is normal; however, the loops are centralized, a sign of free fluid in the peritoneal cavity.

A term male infant weighing 3885 g (>95th percentile) was born vaginally to a 22-year-old woman. No family history of bleeding disorders was reported by the mother. The pregnancy was uneventful and resuscitation was uncomplicated. The initial physical examination in the newborn nursery was unremarkable; however, at 6 hours of age, the infant acutely developed a bluish discoloration to the skin diffusely throughout his body, became hypotensive, and had marked abdominal distention. His hemoglobin was 5.5 g/dL, and his PTT was greater than 100 seconds. A blood transfusion of 20 cc/kg of O Rh-negative packed cells was provided. An abdominal radiograph showed a normal bowel gas pattern, but the intestines were clustered in the center of the abdomen, indicative of free fluid in the peritoneal cavity (Figure 2).

A bedside abdominal ultrasound demonstrated a ruptured splenic hematoma with a large amount of free fluid in the peritoneal cavity. FVIII was <0.01 U/mL, and he responded quickly to repeated intermittent infusions of blood clotting FVIII concentrate. The infant had a rapid recovery and therefore did not require a surgical intervention. He went home on the 10th day of life after abdominal ultrasound confirmed resolution of the splenic laceration and hemoperitoneum. A head ultrasound excluded cranial bleeds.

## Case 3

A large-for-gestational-age, term male infant was born via spontaneous vaginal delivery to a 34-year-old woman. Pregnancy was complicated by little to no prenatal care. Maternal serologies were unknown. The labor was prolonged with rupture of membranes 29 hours before delivery. The infant required oxygen and brief bag mask ventilation with Apgar scores of 4 and 9 at 1 and 5 minutes, respectively. In the first hours of life, a hematoma was noted over the forehead and left parietal area. The CBC done at birth demonstrated a normal hematocrit concentration of 37.6%. The following morning, during his examination, the physician noted the frontal-occipital circumference had increased by 2 cm. The infant also showed signs of increased work of breathing. A repeat blood test revealed a precipitous drop in hemoglobin level from 12.5 g/dL to 8.6 g/dL, and his new hematocrit was 25.7%. FVIII levels were measured at <0.01 U/mL.

**Figure 3.**
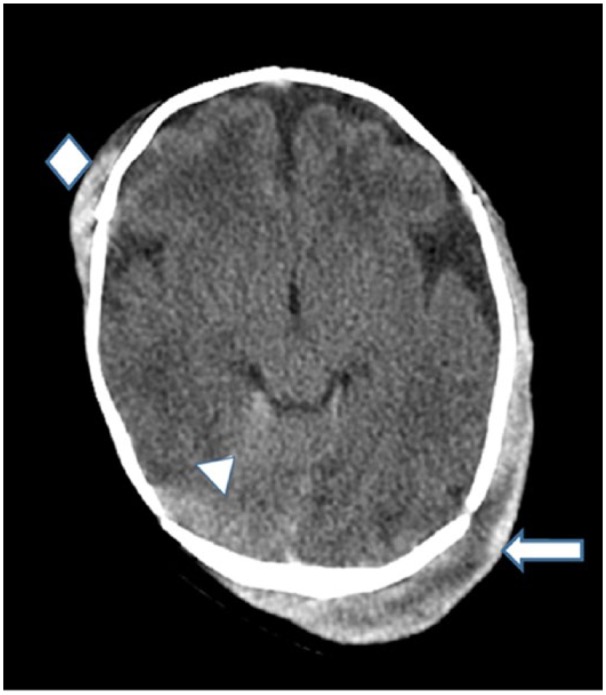
Computed tomography of the head without contrast of infant in case 3. A subdural bleed is seen along the tentorium (arrowhead). A subgaleal hematoma measuring 1.2 cm in thickness involves the left frontoparietal region (arrow). Another subgaleal hematoma measuring 5 mm in thickness is seen in the right frontoparietal region (diamond). No evidence of midline shift.

While awaiting the coagulation profile, he was given 10 mL/kg of fresh frozen plasma and 10 mL/kg of packed red blood cells. The PTT was very elevated, consistent again with severe HA. Hematology was consulted and recommended a continuous infusion of FVIII concentrate until FVIII levels were well over 100%. Meanwhile, a CT of the head showed a subdural hematoma on the right side and a subgaleal hematoma involving the left and right frontoparietal region (Figure 3).

At 36 hours of life, he began having tonic-clonic seizures and was given a loading dose of phenobarbital (20 mg/kg). The electroencephalogram confirmed epileptiform activity. The patient had continuous infusion of the blood clotting factor for 1 week and went home with a central line for daily treatment. The patient was discharged after 3 weeks in the NICU with a magnetic resonance imaging of the head yielding complete resolution of the intracranial and extracranial hemorrhages. The anti-seizure medication was discontinued after 1 week of therapy with a concomitantly normal electroencephalogram. Several days after the infant was born, the mother revealed she knew of a male family member with a bleeding disorder.

## Discussion

Assessing family history of bleeding disorders is crucial for rapid diagnosis, as 50% of all males born to HA-carrying mothers will inherit the disorder. Infants born to known carriers are usually diagnosed early in the neonatal period; however, up to 30% of newborns with this condition may have a negative family history.^5,6^

When evaluating a neonate concerning for HA, one must perform a thorough physical examination and obtain a detailed family history, CBC, coagulation profile, and imaging studies pertinent to examination findings. Patients with hemophilia will present with an age-adjusted elevated PTT and a normal prothrombin time, platelet count, and bleeding time. Ideally, infants who are known to be at risk for hemophilia should have cord blood sent for diagnosis and should avoid intramuscular vitamin K administration.^5^ Prophylactic therapy for children with the severe form of the disease has improved prognosis over the years. Nevertheless, up to 25% of patients with severe hemophilia can suffer from below-average academic performance and behavioral issues, as protracted bleeds can cause developmental and cognitive abnormalities.^7^

Infants with severe hemophilia are prone to spontaneous mouth and head bleeds.^4^ An European study examined the incidence of perinatal bleeding in over 500 hemophiliac infants and found that cranial bleeds were fairly rare, occurring only in 18 children (3.5%)—4 of which resulted in intraparenchymal hemorrhages.^8^ The reported incidence of ICH was higher in a literature review by Kulkarni and Lusher, but the majority of cases were subdural.^6^ Though the incidence of ICH remains low, even within this patient population, approximately half of affected cases may go on to develop seizures, psychomotor retardation, and cerebral palsy.^9^

Additionally, Kulkarni and Lusher found that the most common site of ECH was cephalohematomas. This study examines 2 nonclassic ECH cases with subgaleal hematomas, one of whom sustained a concurrent ICH. Furthermore, both patients survived, in spite of the mortality rate of ICH alone being as high as 20%.^6^ Subgaleal hemorrhages are an uncommon but often lethal condition, which can easily cause significant anemia, hypovolemic shock, and death, even in relatively healthy patients without hemophilia.

A major risk factor for cranial bleeds in hemophilic neonates is vaginal deliveries requiring instrumentation. However, the optimal mode for delivery of an infant at risk for HA remains controversial.^10^ In our cases, the infant with ECH was delivered by caesarean, and the other patients with ICH and visceral bleeds were born vaginally. It is difficult to decipher if the method of delivery played a greater role than the size of the infants, as both patients born vaginally were large for gestation.

Splenic hemorrhage is a rare complication of hemophilia in newborns.^11^ Though there is limited literature regarding this sequela in neonates, it is thought to present in difficult deliveries, large babies, or neonates with severe hemophilia.^8^ Our patient presented with the classic triad of splenic rupture: anemia, abdominal distention, and radiologic findings consistent with hemoperitoneum.^12^ In addition to his severe hemophilia, our patient may have suffered splenic trauma during delivery, given his weight over the 95th percentile.^13^ Although splenic hemorrhages can be identified using an abdominal X-ray, an ultrasound or a CT scan is often used to confirm a laceration. Prompt recognition of hemorrhagic shock along with indicated imaging to confirm visceral bleeding is of utmost importance to prevent mortality and minimize morbidity in affected neonates.

Controversy exists in the treatment of splenic hemorrhage. Our team opted to stabilize our patient’s hemodynamic status first, followed by reevaluation, while maintaining a low threshold for surgical intervention. Fortunately, our patient improved and we did not expose him to the surgical complications seen in postoperative splenectomy patients: chylous/infectious ascites, hematuria, pneumonia, and more bleeding.^12-14^

This case series supplements the current literature by depicting the clinical spectrum of acute, life-threatening hemorrhagic shock in 3 infants with no known family history of hemophilia. The hemorrhages differed in origin and responded quickly to therapy. Once FVIII levels returned to normal, the newborns neither developed any new hemorrhages nor was there an extension of their previous bleed.

In the aforementioned cases, informed consent for patient information to be published was not obtained because this was a retrospective study and the reported data does not include any identifying information.

## Conclusion

Although hemophilia commonly presents in the neonatal period with bleeding after circumcision or venipuncture, neonatal providers should be aware that HA can be life-threatening and that up to one third of newborns diagnosed will have a negative family history.^6^ Prognosis depends on early recognition, immediate volume resuscitation, and administration of recombinant FVIII.
